# Spontaneous Recovery of Upper Extremity Motor Impairment After Ischemic Stroke: Implications for Stem Cell-Based Therapeutic Approaches

**DOI:** 10.1007/s12975-017-0523-9

**Published:** 2017-02-15

**Authors:** Hossein Delavaran, Joseph Aked, Håkan Sjunnesson, Olle Lindvall, Bo Norrving, Zaal Kokaia, Arne Lindgren

**Affiliations:** 1 Department of Clinical Sciences Lund, Division of Neurology, Lund University, Lund, Sweden; 2 Department of Neurology and Rehabilitation Medicine, Skåne University Hospital, Lund, Sweden; 3 Center for Medical Imaging and Physiology, Skåne University Hospital, Lund, Sweden; 4 Laboratory of Stem Cells and Restorative Neurology, Lund Stem Cell Center, Lund University, Lund, Sweden

**Keywords:** Stroke, Upper extremity motor impairment, Recovery, Stem cell therapy

## Abstract

Preclinical studies suggest that stem cell therapy (SCT) may improve sensorimotor recovery after stroke. Upper extremity motor impairment (UEMI) is common after stroke, often entailing substantial disability. To evaluate the feasibility of post-stroke UEMI as a target for SCT, we examined a selected sample of stroke patients potentially suitable for SCT, aiming to assess the frequency and recovery of UEMI, as well as its relation to activity limitations and participation restrictions. Patients aged 20–75 years with first-ever ischemic stroke, and National Institutes of Health Stroke Scale (NIHSS) scores 1–18, underwent brain diffusion-weighted MRI within 4 days of stroke onset (*n* = 108). Survivors were followed up after 3–5 years, including assessment with NIHSS, Fugl-Meyer assessment of upper extremity (FMA-UE), modified Rankin Scale (mRS), and Stroke Impact Scale (SIS). UEMI was defined as NIHSS arm/hand score ≥1. UEMI recovery was evaluated with change in NIHSS arm/hand scores between baseline and follow-up. Of 97 survivors, 84 were available to follow-up. Among 76 subjects (of 84) without recurrent stroke, 41 had UEMI at baseline of which 10 had residual UEMI at follow-up. The FMA-UE showed moderate-severe impairment in seven of 10 survivors with residual UEMI. UEMI was correlated to mRS (*r*_s_ = 0.49, *p* < 0.001) and the SIS social participation domain (*r*_s_ = −0.38, *p* = 0.001). Nearly 25% of the subjects with UEMI at baseline had residual impairment after 3–5 years, whereas about 75% showed complete recovery. Most of the subjects with residual UEMI had moderate-severe impairment, which correlated strongly to dependency in daily activities and social participation restrictions. Our findings suggest that SCT targeting post-stroke UEMI may be clinically valuable with significant meaningful benefits for patients but also emphasize the need of early prognostication to detect patients that will have residual impairment in order to optimize patient selection for SCT.

## Introduction

Stem cell therapy (SCT) has emerged as a potential therapeutic option for functional restoration after the acute phase of stroke [1]. Effects of tissue-specific neural stem cells and non-tissue-specific mesenchymal stem cells have been associated with improved functional recovery in animal models of stroke, mediated through mechanisms such as trophic effects, modulation of inflammation, neuroprotection, stimulation of angiogenesis, and possibly by neuronal replacement [2, 3]. Clinical stroke trials with SCT are ongoing, mostly investigating safety in limited numbers of selected patients [3–6]. However, the efficacy of SCT for stroke patients remains to be demonstrated, and the choice of which outcome variables to study is a crucial issue for the optimal design of later-phase pivotal trials.

Stroke lesion sizes and locations are highly heterogeneous, and the clinical manifestations of stroke are also diverse encompassing a broad variety of neurological impairments and varying degrees of severity. Correspondingly, there is much inter-individual variation in functional recovery after stroke [7]. Furthermore, the rates and extent of recovery also differ depending on the type of neurological impairment [8]. Consequently, SCT may have varying effects on different aspects of functional recovery after stroke. To demonstrate the maximum potential treatment effect, it has therefore been suggested that domain-specific outcomes may be best suited as end-points in stroke trials aiming to study the efficacy of SCT [8].

Recovery of impaired motor functions after stroke, such as recovery of upper extremity motor impairment (UEMI), may be one such well-defined domain-specific outcome. UEMI is especially interesting in this regard as preclinical SCT studies have mainly focused on recovery of sensorimotor functions after stroke [3, 8], and behavioral assessments in rodent stroke models have demonstrated that SCT can significantly improve forelimb motor function [9–12]. In humans, UEMI can be a major consequence following stroke and may entail substantial disability causing dependency in activities of daily living (ADLs) and reduced health-related quality of life (HRQoL) [13–15]. In addition, UEMI can readily be evaluated objectively with commonly used clinical assessment measures [16]. However, the feasibility of UEMI as a target for SCT in stroke patients is unclear.

We therefore examined a selected group of ischemic stroke patients, potentially suitable for SCT, assessing (i) the proportion and characteristics of subjects with UEMI, (ii) the degree of spontaneous recovery of UEMI during a 3–5-year period after stroke, and (iii) the relation of UEMI to activity limitations and participation restrictions after stroke.

## Methods

### Sample

The study sample (*n* = 108) was recruited from a consecutive series of first-ever ischemic stroke patients admitted to Skåne University Hospital in Lund, Sweden, in 2009–2011. Stroke was defined by the World Health Organization (WHO) criteria [17]. CT of the head was performed on all patients to exclude intracranial hemorrhage.

To select stroke patients potentially suitable for SCT, patients were prospectively included at baseline if they were aged 20–75 years, had a National Institutes of Health Stroke Scale (NIHSS) score of 1–18 on days 2–4 after stroke onset [18], could perform diffusion-weighted MRI (DW-MRI) within 4 days of stroke onset, and gave written informed consent to participate. Exclusion criteria were symptoms or CT findings strongly suggestive of brainstem or cerebellar infarction, severe concomitant disease, or contraindications to MRI. Details on case ascertainment at baseline, as well as baseline assessments and variables, have been previously described [19].

### Follow-up Procedure

Clinical follow-up examination was performed by a physician (blinded to the baseline data) for each participating stroke survivor 3–5 years after stroke onset at the outpatient clinic of the Department of Neurology and Rehabilitation Medicine at Skåne University Hospital. Stroke survivors unable to come to the outpatient clinic were offered follow-up through home visit.

### Clinical Assessments

The clinical assessment protocol at follow-up conformed to the WHO International Classification of Functioning, Disability and Health [20], including evaluations at the levels of body functions and structures, activity, and participation, as follows:

1. Body functions and structures

The NIHSS was used to measure the severity of stroke symptoms [18]. Similar to previous studies, we also included the assessment of distal upper limb motor function by adding the additional item for motor function in the hand (NIHSS item 12; scores 0–2) to the official NIHSS (composite scores 0–46) [21]. Overall stroke severity was stratified according to the NIHSS scores as follows: no symptoms = 0, mild = 1–4, moderately severe = 5–14, and severe = ≥15 [22].

UEMI was defined as a score of ≥1 on the combined NIHSS arm and hand items (composite scores 0–6). In case of bilateral UEMI, the mostly impaired side was evaluated throughout the study. The NIHSS was also used for the longitudinal assessment of the recovery of UEMI between baseline and follow-up (ΔNIHSS arm/hand). The Fugl-Meyer Assessment of Upper Extremity (FMA-UE, scores 0–66) was used for more detailed evaluation of the stroke survivors with UEMI [23]. FMA-UE consists of 33 items that are divided into the following subsections: shoulder-arm (scores 0–36), wrist (scores 0–10), hand (scores 0–14), and upper limb coordination (scores 0–6) [23]. The degree of UEMI was defined on the basis of FMA-UE scores as follows: severe = 0–22, moderate = 23–52, and mild = 53–66 [24, 25].

2. Activity

The modified Rankin Scale (mRS, scores 0–5) was used to assess the degree of functional independence in ADL with reference to pre-stroke activities [26]. In addition, the upper extremity functioning at the level of activities was evaluated with the Action Research Arm Test (ARAT, scores 0–57) [27, 28]. The ARAT contains 19 items grouped into the following subtests: grasp (scores 0–18), pinch (scores 0–12), grip (scores 0–18), and gross movement (scores 0–9) [27, 28]. The upper extremity functional capacity was defined according to the following ARAT cut-points as follows: none = 0–10, poor = 11–21, limited = 22–42, notable = 43–54, or full = 55–57 [24].

3. Participation

HRQoL was assessed using the Stroke Impact Scale (SIS), version 2.0, which evaluates the patient-reported impact of stroke on different domains of health and life including ADL and social participation [29]. The first question of the Short-Form 36 Health Survey (SF-36) was used to assess the patient-reported overall health status (“In general, would you say your health is: excellent, very good, good, fairly good, or poor?”) [30].

### Imaging and Image Interpretation

The MR imaging procedure at baseline has been previously described [19]. In summary, all patients were examined with transversal DW-MRI as well as transversal FLAIR-, GRE-, and sagittal T2-weighted sequences within 4 days of stroke onset. A neuroradiologist blinded to the clinical information analyzed the images. Extent of acute focal ischemic lesions was defined as the area of restricted diffusion on diffusion-weighted sequences. For stroke patients with UEMI, we also assessed the ischemic lesions’ possible involvement of the motor cortex including the primary motor cortex, the premotor areas, and the supplementary motor areas. Likewise, we assessed the possible involvement of the corticospinal tract by estimating the location of the corticospinal tract on MR imaging using previously established neuroradiological methods [31].

To illustrate the ischemic lesions in stroke patients with UEMI, a lesion overlap map was created. The identified lesions on the DW-MRI sequences were manually drawn on the closest corresponding slices of the ICBM 2009a Nonlinear Symmetric 1 × 1 × 1 mm T1-weighted template (supplied by McConnell Imaging Center, Montreal, Canada), using MRIcron software [32, 33]. In order to simplify the overlap map, the lesions were oriented such that the clinically relevant stroke lesions were assumed to be in the left hemisphere. The lesion overlap plot was then obtained using the MRIcron software.

### Statistical Analysis

The Mann-Whitney two-sample test and Fisher’s exact test were used for comparisons with continuous and categorical variables, respectively. Spearman’s rank-order correlation was used to evaluate the relation of UEMI (defined as NIHSS arm/hand score ≥1) to the degree of independence in ADL as measured with mRS, HRQoL as assessed with the SIS domains regarding ADL and social participation, as well as with the first question of SF-36 regarding overall health status. *p* values <0.05 were considered significant. The statistical calculations were performed using SPSS software (version 22, released 2013; IBM SPSS Statistics for Windows, IBM Corp, Armonk, NY).

## Results

**Fig. 1:**
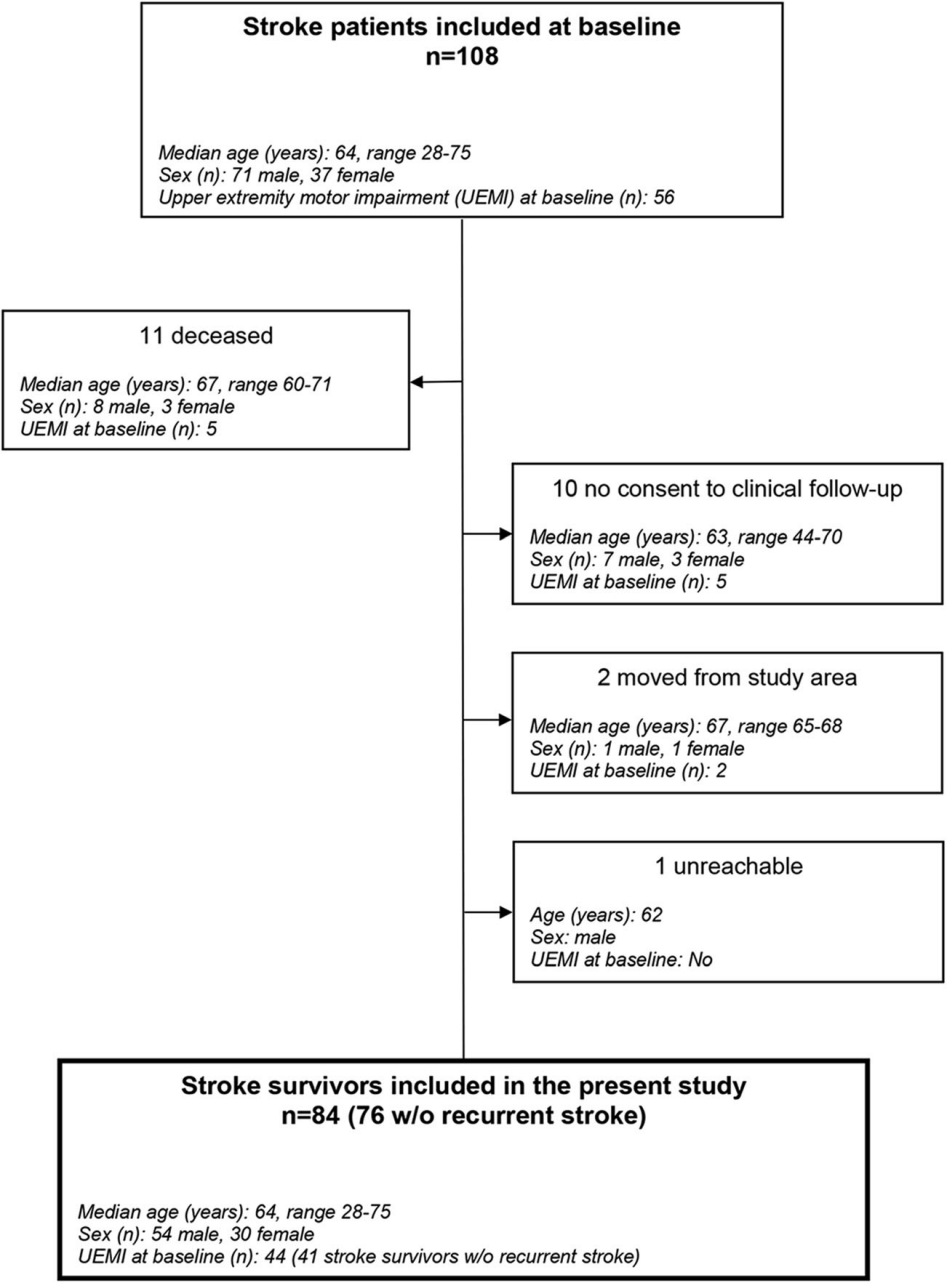
Patient flowchart. The age indicated is age at stroke onset

Of the 108 stroke patients included at baseline, 11 (10%) were deceased at the time of follow-up. In total, 84 (87%) of the 97 stroke survivors performed the clinical follow-up examination. The dropout reasons and sample flow from baseline to follow-up are illustrated in Fig. 1, and the details on demographic and baseline characteristics of the included stroke survivors versus the deceased stroke patients are presented in Table 1.

**Table 1:** Demographics and baseline characteristics for included ischemic stroke patients

Variable	Survivors (*n* = 84)	Deceased before follow-up^a^ (*n* = 11)
Sex, *n* (%)		
Female	30 (36)	3 (27)
Age at stroke onset, median (range)	64 (28–75)	67 (60–71)
Acute recanalization treatment^b^, *n* (%)	13 (15)	1 (9)
NIHSS on days 2–4 after stroke onset, median (range)	3 (1–18)	3 (1–7)
UEMI on days 2–4 after stroke onset, *n* (%)	44 (52)	5 (46)

*NIHSS* National Institutes of Health Stroke Scale, including hand item 12 (composite scores 0–46); *UEMI* upper extremity motor impairment, defined as the NIHSS arm/hand score of ≥1

^a^The median time from index stroke to death was 31 months (range 0–57)

^b^Thrombolysis and/or endovascular treatment

### Characteristics of Stroke Patients at Follow-up

The median age of the 84 included stroke survivors was 68 years (range 33–80), and 30 (36%) of these were female. The median time from stroke onset to follow-up was 4.6 years (range 3.5–5.7). In total, 41/84 stroke survivors had received some form of rehabilitation therapy after the index stroke, with a median time of rehabilitation of 5 weeks (range 1–109). Recurrent strokes were reported for 8/84 stroke survivors. Further details on follow-up characteristics of the included stroke survivors are presented in Table 2.

**Table 2:** Follow-up characteristics of the included ischemic stroke survivors

Variable	Survivors without recurrent stroke (*n* = 76)	Survivors with recurrent stroke (*n* = 8)
Sex, *n* (%)		
Female	26 (34)	4 (50)
Age at follow-up, median (range)	68 (33–80)	72 (62–77)
Stroke severity^a^, *n* (%)		
No symptoms	40 (53)	2 (25)
Mild	31 (41)	4 (50)
Moderately severe	5 (7)	2 (25)
Severe	0	0
Overall disability^b^, *n* (%)		
No/slight	71 (93)	5 (63)
Moderate	4 (5)	1 (13)
Severe	1 (1)	2 (25)

^a^Overall stroke severity was classified according to the National Institutes of Health Stroke Scale including hand item 12 (NIHSS, composite scores 0–46), as follows: no symptoms = 0, mild = 1–4, moderately severe = 5–14, and severe = ≥15

^b^Overall disability was classified according to the modified Rankin Scale (mRS, scores 0–5), as follows: no/slight = 0–2, moderate = 3, and severe = 4–5

### Frequency and Characteristics of Stroke Patients with UEMI

**Fig. 2:**
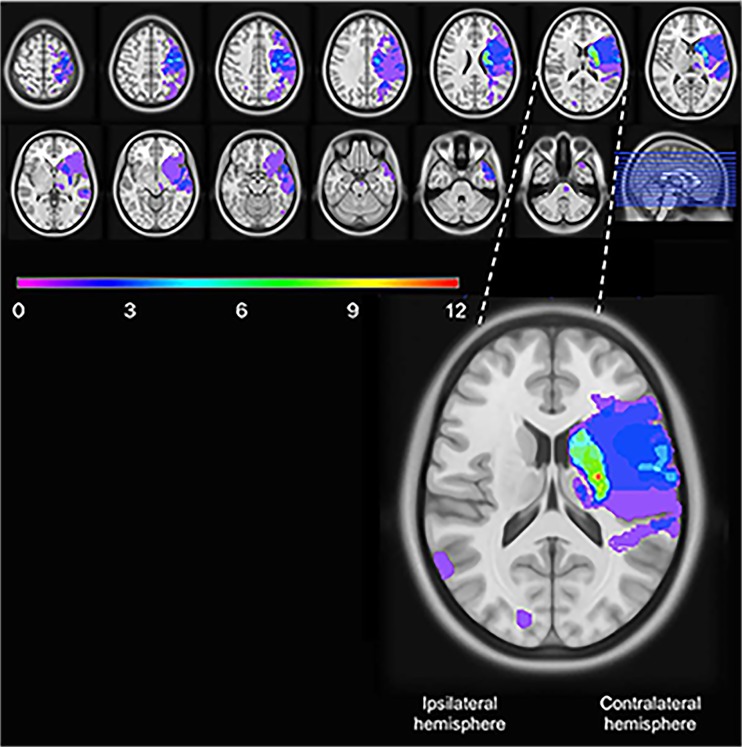
Lesion overlap image of the stroke survivors with upper extremity motor impairment at baseline (*n* = 41 stroke survivors without recurrent stroke). The lesions were oriented such that the clinically relevant stroke lesions were assumed to be in the left hemisphere. The *color bar* indicates the number of overlapping lesions, and the *enlarged picture* shows the slice with the maximum number of overlapping lesions

Among the 76 stroke survivors with no recurrent stroke, 41 had UEMI at baseline of whom 10 displayed residual UEMI after 3–5 years. Analysis of the DW-MRIs showed corticosubcortical infarcts in 14/41 stroke survivors with UEMI at baseline, all involving the motor cortex and/or the estimated course of the corticospinal tract. Sole cortical infarcts were observed in 3/41 stroke survivors, all with motor cortex involvement. Moreover, only subcortical infarcts were seen in 19/41 stroke survivors, and the estimated course of the corticospinal tract was involved in 12 of these cases. In addition, 2/41 stroke survivors had brainstem infarcts (without clear localizing symptoms from the brainstem), and 3/41 had MRI negative strokes. The MRI overlap image showing the brain infarcts of the stroke survivors with UEMI at baseline is presented in Fig. 2.

### Spontaneous Recovery of UEMI in Stroke Patients

**Fig. 3:**
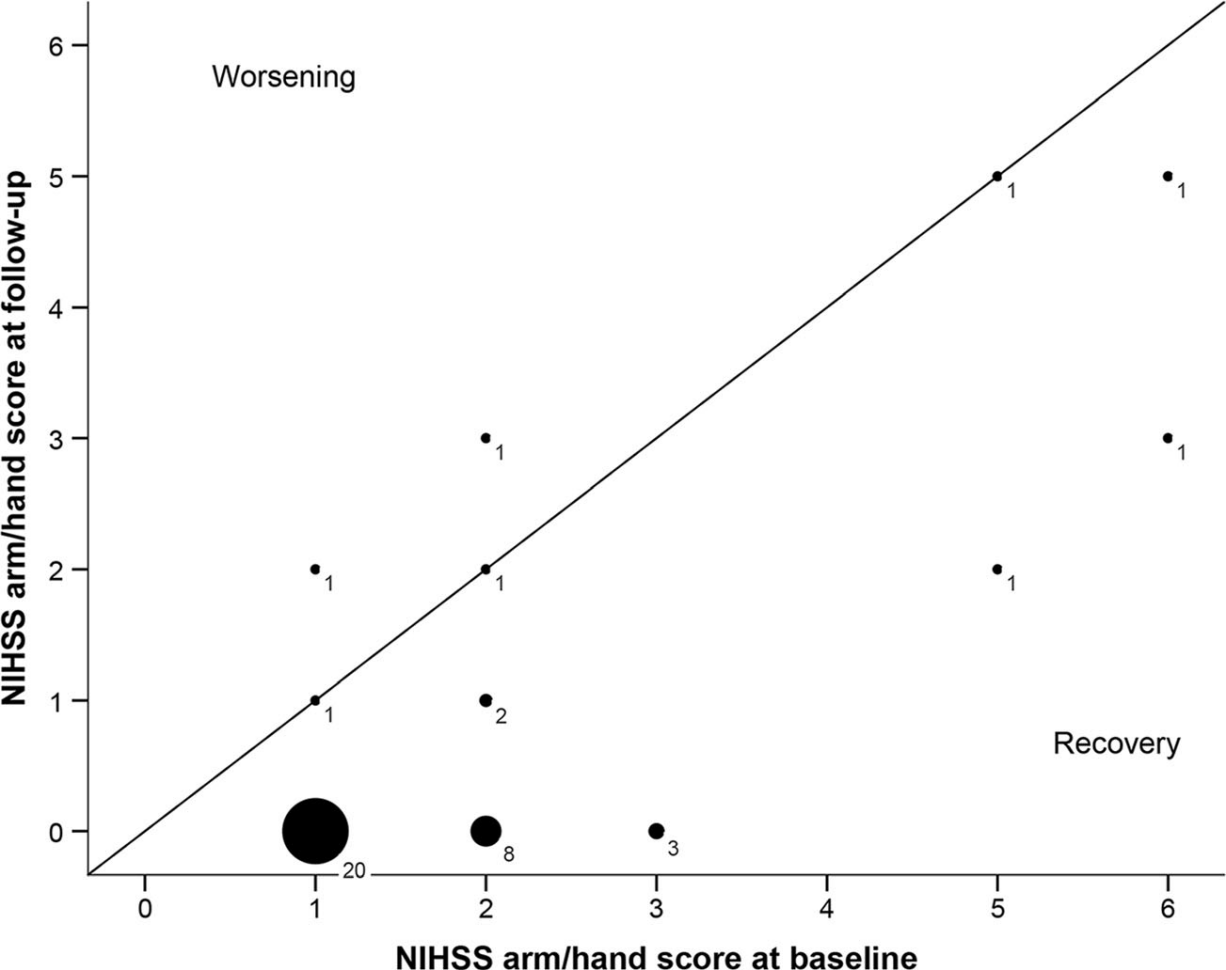
Bubble plot illustrating the spontaneous recovery of upper extremity motor impairment (*n* = 41 stroke survivors without recurrent stroke), defined as a change in scores on the combined arm/hand items of the National Institutes of Health Stroke Scale between baseline and follow-up (ΔNIHSS arm/hand). Median ΔNIHSS arm/hand = −1 (range −3 to 1). The *bubble size* indicates the number of subjects

Among the stroke survivors with UEMI at baseline, 31/41 showed complete recovery at follow-up. Of the 10/41 stroke survivors with residual UEMI at follow-up, 5/10 displayed only partial recovery whereas the other 5/10 showed no recovery at all. Of these 10 individuals with residual UEMI, 3 had a mild degree of residual impairment (FMA-UE = 53–66), 4 had a moderate degree of residual impairment (FMA-UE = 23–52), and 3 had severe residual impairment (FMA-UE = 0–22). The spontaneous recovery of UEMI is illustrated in Fig. 3.

The stroke survivors with partial or no UEMI recovery had larger lesion volumes (*p* = 0.003) and higher NIHSS scores at baseline (*p* < 0.001) as compared to the stroke survivors with complete UEMI recovery (Table 3). Also, the motor cortex was more frequently involved among the survivors with worse UEMI recovery (*p* = 0.03) (Table 3).

**Table 3:** Characteristics of the included ischemic stroke survivors with no or partial UEMI recovery versus those with complete recovery

Variable	No or partial UEMI recovery (*n* = 10)	Complete UEMI recovery (*n* = 31)	*p* value
Sex, *n* (%)			
Female	5 (50)	10 (32)	ns^a^
Age at stroke onset, median (range)	65 (36–74)	64 (28–75)	ns^b^
NIHSS at baseline, median (range)	7 (2–18)	3 (1–8)	<0.001^b^
NIHSS arm/hand at baseline, median (range)	2 (1–6)	1 (1–3)	0.004^b^
Lesion volume in mL, median (range)	26.5 (0.4–155.3)	1.0 (0.1–23.3)	0.003^b^
Lesion location, *n* (%)			
Cortical only	0	3 (11)	ns^a^
Subcortical only	3 (30)	16 (57)	ns^a^
Corticosubcortical	7 (70)	7 (25)	0.02^a^
Motor cortex involvement, *n* (%)	7 (70)	8 (29)	0.03^a^
Corticospinal tract involvement, *n* (%)	8 (80)	18 (64)	ns^a^

*NIHSS* National Institutes of Health Stroke Scale, including hand item 12 (composite scores 0–46); *UEMI* upper extremity motor impairment, defined as the NIHSS arm/hand score of ≥1

*ns* not significant

^a^Fisher’s exact test

^b^The Mann-Whitney two-sample test

### Activity Limitations and Participation Restrictions in Stroke Patients with UEMI

The assessment of functional independence in ADL with mRS among the 10 individuals with residual UEMI at follow-up showed that 7 had no/slight disability (mRS = 0–2), 2 had moderate disability (mRS = 3), and 1 had severe disability (mRS = 4–5).

With regard to the functional capacity of the impaired upper extremity, 3/10 had full capacity (ARAT = 55–57), 1/10 had notable capacity (ARAT = 43–54), 1/10 had limited capacity (ARAT = 22–42), whereas 1/10 had poor capacity (ARAT = 11–21) and 4/10 showed no functional capacity (ARAT = 0–10).

In total, 9/10 individuals with residual UEMI reported problems in ADL and all 10 of them described difficulties in social participation as assessed with the SIS. However, the overall health status was described as “very good” by 1/10 individuals with residual UEMI, as “good” by 3/10, and “fairly good” by 4/10, while 2/10 described it as “poor.”

**Fig. 4:**
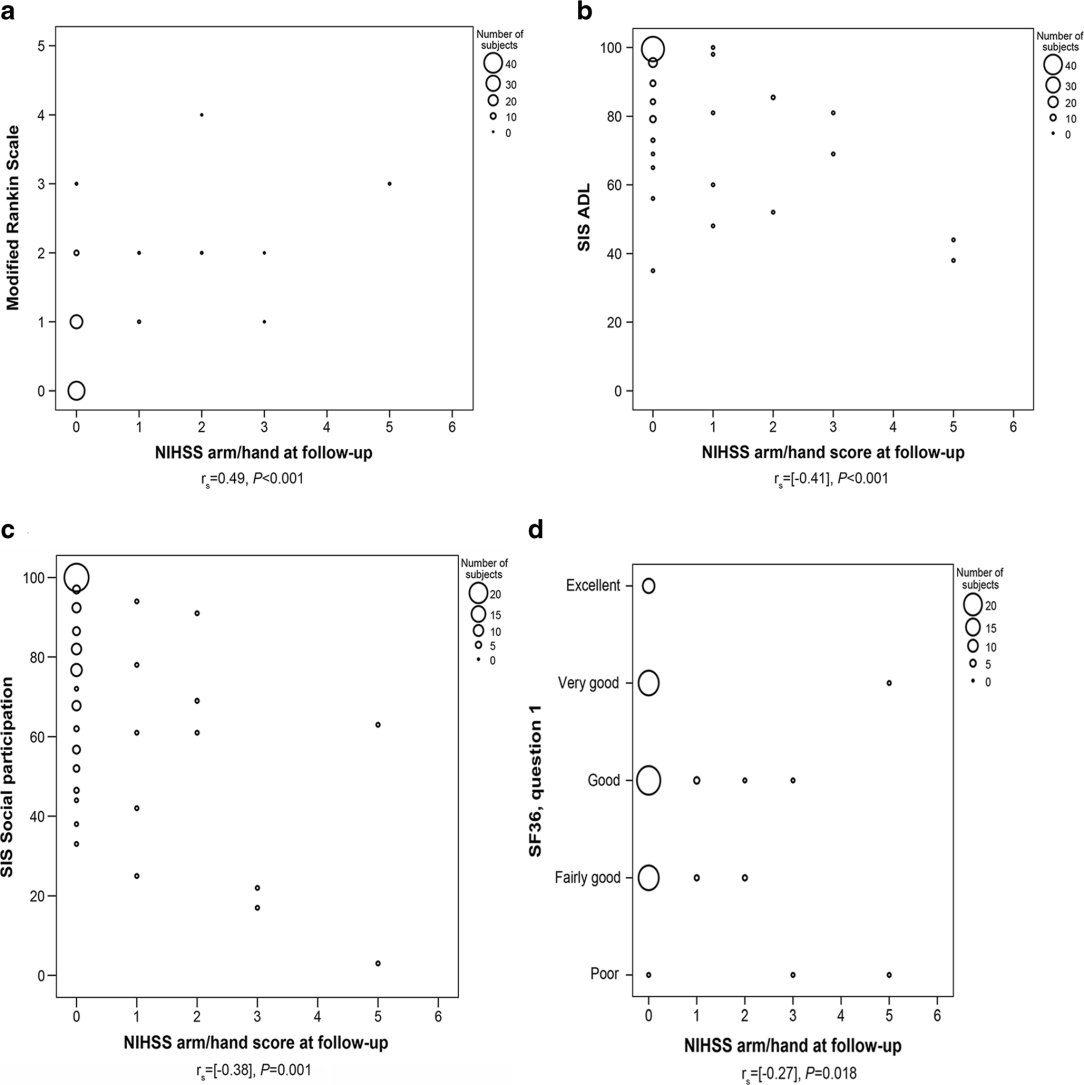
Scatter plots illustrating the correlation of UEMI, as measured with scores on the combined arm/hand items of the National Institutes of Health Stroke Scale at follow-up (*n* = 76 stroke survivors without recurrent stroke), to **a** dependency in daily activities according to the modified Rankin Scale (mRS), **b** dependency in daily activities according to Stroke Impact Scale (*SIS*) item 5, **c** social participation restrictions according to SIS item 8, and **d** overall health status according to the first question of Short-Form 36 Health Survey (*SF-36*)

Moreover, UEMI correlated to dependency in ADL as evaluated with mRS (*r*_s_ = 0.49, *p* < 0.001). Likewise, UEMI was also correlated to the SIS domains concerning ADL (*r*_s_ = −0.41, *p* < 0.001) and social participation (*r*_s_ = −0.38, *p* = 0.001), as well as to the patient-reported overall health status as assessed by SF-36 (*r*_s_ = −0.27, *p* = 0.018) (Fig. 4).

## Discussion

Our study shows that UEMI was prevalent in this selected group of ischemic stroke patients potentially suitable for SCT, as roughly 50% had UEMI in the first days post stroke. Moreover, nearly 25% of the subjects with UEMI at baseline had residual impairment after 3–5 years of which half displayed either poor or no upper extremity functional capacity. However, there was considerable variability in the spontaneous recovery of UEMI and about 75% of the subjects with UEMI at baseline eventually recovered completely. In addition, UEMI was strongly correlated to dependency in daily activities and participation restrictions after stroke.

The primary patient selection objective for the present study aimed for detecting potential candidates for SCT. To the best of our knowledge, this is the first study to report the frequency of UEMI in such a selected group of ischemic stroke patients. Nearly 52% of the patients in our study displayed UEMI in the first days after stroke. Previous community- and population-based stroke studies have reported a high prevalence of upper limb motor deficits in the acute phase, ranging between approximately 70 and 80% [34, 35]. Another community-based study described a prevalence of 45% regarding upper limb motor dysfunction after 6 months post stroke [36]. Most of these previous epidemiological studies on post-stroke UEMI were performed several years (and sometimes decades) ago [34–36]. A more recent hospital-based study reported that 48% of the patients in a non-selected group with first-ever stroke had UEMI within 72 h of onset [37]. However, the comparison between studies is difficult because of different study designs, assessment methods, and case mix. Nevertheless, our findings of a high proportion of subjects with UEMI in this selected group of ischemic stroke patients are in accordance with the previous studies reporting a high prevalence of UEMI after stroke.

Our study also highlights the variability in spontaneous recovery of UEMI after stroke, as many stroke patients displayed a complete recovery whereas some only had partial recovery and others had none (Fig. 3). This is consistent with previous studies which have shown considerable inter-individual variability in the course and degree of spontaneous recovery of post-stroke UEMI [7, 38]. To assess determinants of UEMI recovery was beyond the scope of this study due to the limited size of the study cohort. Nevertheless, there seemed to be a trend towards a higher degree of initial paresis in the arm/hand, larger lesion volumes, as well as higher frequency of motor cortex involvement among the stroke patients with partial or no UEMI recovery as compared to those with complete recovery. In line with these findings, previous studies have reported that important predictors of UEMI recovery include not only initial degree of severity of paresis and lesion size but also lesion location as well as the extent of injury on descending motor pathways, age, and comorbid medical conditions including depression and cognitive impairment [38–43]. Also, advanced neuroimaging and electrophysiological studies have shown that functional and structural changes in the perilesional brain tissue as well as in more extensive bi-hemispheric networks are important biomarkers of recovery potential after stroke [43]. Additionally, genetic factors such as alterations in the brain-derived neurotrophic factor gene may influence brain plasticity and recovery after stroke [44].

Furthermore, our results demonstrate the significance of post-stroke UEMI as it correlates strongly to dependency in ADL, patient-reported social participation restrictions, and patient-reported overall health status. This is also consistent with previous studies reporting that impaired motor functions, and UEMI in particular, are important contributory factors in stroke patients’ dependency in daily activities [15]. However, even though 70% of the individuals with residual UEMI in our study had moderate-severe impairment at follow-up and 60% of them had either limited, poor, or no upper extremity functional capacity, some of these individuals described their overall health status in positive terms. As previously reported, this indicates that aspects other than impairments and disabilities may also be important for self-perceived health and HRQoL [45, 46], and emphasizes the importance of using both objective and patient-reported assessment measures when evaluating UEMI after stroke [47].

Taken together, our findings have several important implications for the application of SCT in stroke patients. Firstly, the high frequency of UEMI during the first days after stroke, the non-negligible proportion of patients with poor spontaneous recovery and substantial residual impairment long-term after stroke, as well as the strong correlation of UEMI to activity limitations and participation restrictions suggest that SCT targeting post-stroke UEMI may be clinically valuable with significant meaningful benefits for patients. Secondly, the observed variability in spontaneous recovery of UEMI, and the fact that roughly three quarters of the patients with UEMI recovered completely, emphasizes the necessity of early prognostication of patients that will have residual impairment and will be in need of a recovery-promoting treatment. From the perspective of SCT, the ability to stratify stroke patients on the basis of predicted potential for recovery will be central for patient selection. Although certain predictors of UEMI recovery after stroke have been previously reported, as mentioned above, predicting such recovery in individual stroke patients is difficult. An algorithm has recently been proposed to predict individual stroke patients’ potential for functional recovery of UEMI within 3 months after stroke and combines data from clinical assessments in the acute phase (shoulder abduction and finger extension at 72 h after symptom onset), neurophysiological examination (transcranial magnetic stimulation), and neuroimaging (DW-MRI) [48]. Another recent study reported that scorings on two items on the ARAT, assessed at 3 days from stroke onset, predicted the upper extremity function required for a drinking task with high accuracy during the first year after stroke [49]. However, further studies are needed to determine the feasibility and validity of these algorithms. Lastly, our findings suggest that UEMI recovery is a clinically relevant and feasible domain-specific outcome that may be suitable as an end-point in stroke trials aiming to study the efficacy of SCT. In addition to using established objective assessment measures of UEMI, we recommend that such trials also include patient-reported outcome measures to better reflect the patients’ perspectives with regard to their impairments and subsequent disabilities. Hence, multiple assessment measures of UEMI could be included as a composite modality-specific end-point that takes into account the different dimensions of post-stroke UEMI, including loss of body functions and structures (as measured with, e.g., FMA-UE), activity limitations (as measured with, e.g., ARAT), and participation restrictions (as evaluated with, e.g., SIS or SF-36).

It should be noted, though, that there are limitations with our study. One limitation is the hospital-based case ascertainment which does not allow for a more accurate estimate of the prevalence of post-stroke UEMI. Also, the patient selection criteria in our study entail restrictions for a more precise estimate of the overall prevalence of UEMI after stroke. However, given the strict inclusion and exclusion criteria, our aim was to study UEMI in this selected group of stroke patients initially deemed to be potential SCT candidates. Moreover, many included patients in the original study sample had mild stroke symptoms with relatively low overall NIHSS scores. It is currently unclear if such patients will be realistic candidates for SCT. Nonetheless, even low overall NIHSS scores may represent significant disability causing lowered HRQoL. Our study is also limited by the relatively low number of participants and a 12% loss to follow-up (Fig. 1) which may have caused a selective loss of patients with residual UEMI and subsequent underestimation of the long-term post-stroke UEMI frequency. Furthermore, there were no interim assessments of the stroke patients in the period between baseline and follow-up which might have given additional and more detailed information about the natural history of spontaneous recovery of UEMI. Besides, nearly half of the patients had received some form of targeted rehabilitation, aiming to promote the recovery process and restore function, but we could not evaluate the effects of rehabilitation as this was not assessed in more detail. Finally, we defined UEMI according to impaired performance on the NIHSS arm and/or hand items which may have underestimated the frequency of UEMI. The use of a more comprehensive assessment measure such as the FMA-UE, to define UEMI at both baseline and follow-up, might have yielded different results. It could also be argued that the addition of the unvalidated hand item to the official NIHSS contributes little to the measurement of the structures underlying the NIHSS [50], but assessment of distal upper limb motor function in addition to proximal upper limb motor function may be of value.

In conclusion, SCT targeting post-stroke UEMI may be clinically valuable with significant meaningful benefits for patients since UEMI is frequent after stroke and correlates strongly to dependency in daily activities and social participation restrictions. Our findings also demonstrate considerable inter-individual variability in spontaneous recovery of post-stroke UEMI, which emphasizes the necessity of validated prognostication models that enable early stratification of patients that will have residual impairment and will be in need of recovery-promoting treatments such as SCT.
